# Increased Expression of Ephrins on Immune Cells of Patients with Relapsing Remitting Multiple Sclerosis Affects Oligodendrocyte Differentiation

**DOI:** 10.3390/ijms22042182

**Published:** 2021-02-22

**Authors:** Maya Golan, Avivit Krivitsky, Karin Mausner-Fainberg, Moshe Benhamou, Ifat Vigiser, Keren Regev, Hadar Kolb, Arnon Karni

**Affiliations:** 1 The Neuroimmunology and Multiple Sclerosis Unit, Neurology Institute, Tel Aviv Sourasky Medical Center, Tel Aviv 64239, Israel; mayagola@gmail.com (M.G.); avivitkr7@gmail.com (A.K.); fainbergkarin@gmail.com (K.M.-F.); moshe.bh@gmail.com (M.B.); ifatv@tlvmc.gov.il (I.V.); kerenre@tlvmc.gov.il (K.R.); hadarko@tlvmc.gov.il (H.K.); 2 Sackler School of Medicine, Tel Aviv University, Tel Aviv 69978, Israel; 3 Sagol School of Neuroscience, Tel Aviv University, Tel Aviv 69978, Israel

**Keywords:** multiple sclerosis, T cells, ephrins, oligodendrocyte precursor cells, oligodendrocyte differentiation

## Abstract

The effect of the inflammatory response on regenerative processes in the brain is complex. This complexity is even greater when the cause of the tissue damage is an autoimmune response. Multiple sclerosis (MS) is an immune-mediated disease in which demyelination foci are formed in the central nervous system. The degree of repair through oligodendrocyte regeneration and remyelination is insufficient. Ephrins are membrane-bound ligands activating tyrosine kinase signaling proteins that are known to have an inhibitory effect on oligodendrocyte regeneration. In this study, we examined the expression of ephrins on immune cells of 43 patients with relapsing-remitting (RR) MS compared to 27 matched healthy controls (HC). We found an increased expression of ephrin-A2, -A3 and -B3, especially on T cell subpopulations. We also showed overexpression of ephrins on immune cells of patients with RR-MS that increases the forward signaling pathway and that expression of ephrins on immune cells has an inhibitory effect on the differentiation of oligodendrocyte precursor cells (OPCs) in vitro. Our study findings support the concept that the immune activity of T cells in patients with RR-MS has an inhibitory effect on the differentiation capacity of OPCs through the expression and forward signaling of ephrins.

## 1. Introduction

Multiple sclerosis (MS) is an immune-mediated disease of the central nervous system that causes lesions of demyelination along with damage to oligodendrocytes. The disease pathology also affects the axons and neurons, with both direct and secondary damage attributed to the demyelination [1,2,3,4]. The repair of the myelin sheath in the MS lesions (remyelination) is essential for recovery. However, this repair is usually limited. The MS lesions may be populated by oligodendrocyte precursor cells (OPCs) [5,6,7,8,9], which are derived from the subventricular zone (SVZ) [10,11] and the white matter parenchyma [12], and they may regenerate into myelinating oligodendrocytes [13,14] and even extensively in some cases [15,16]. However, OPCs differentiation is usually insufficient to produce mature myelinating oligodendrocytes in demyelinating lesions of MS [6,9,17,18,19], and the lesions are replaced by large numbers of reactive astrocytes that form a nonfunctional glial scar [20,21,22].

It has been recently implicated that the immune system plays a pivotal role during tissue repair and regeneration [23,24,25]. Being as it is an immune-mediated disease, it is important to understand the effect of inflammation on the regenerative capacity of oligodendrocytes and on remyelination in MS. A negative correlation between the presence of inflammatory infiltration and the degree of remyelination was reported in MS [6]. In addition, it was found that different inflammatory factors and cells have different effects upon myelin repair. The innate immune activity of macrophages and microglia may support or prevent remyelination [26,27,28,29,30], while the adaptive activity of T lymphocytes tends to prevent oligodendrogenesis and remyelination. The proinflammatory Th1 and Th17 subsets of T cells had toxic effects on OPCs in vitro [29] and reduced remyelination in vivo in a cuprizone model of demyelination [30]. However, the existence of a pro-regenerative subset of T cells has been suggested since the depletion of CD4^+^ or CD8^+^ T cells led to an impairment of remyelination [31]. Moreover, it was recently reported that regulatory T cells (Tregs) directly promote myelin regeneration in the CNS [32,33,34]. However, T cells are also implicated in the neurodegeneration that occurs in MS since different cytokines secreted by CD4^+^ and CD8^+^ T cells sensitize glutamate (excitotoxic) receptors and increase glutamate excitotoxicity [35,36,37].

Molecular factors originating from inflammation are known to affect the differentiation of oligodendrocytes since supernatants of activated peripheral blood mononuclear cells (PBMCs) and, especially, of CD4^+^ T cells, significantly inhibited oligodendroglial differentiation [38]. Interferon-γ, the hallmark cytokine of Th1 cells, inhibited OPCs differentiation [39]. We had found that the immune cells of patients with RR-MS produced increased levels of factors that inhibit oligodendrogenesis, such as bone morphogenic proteins, and reduced levels of factors, such as noggin, follistatin, DAN and coco that support oligodendrogenesis [40,41,42,43,44].

Ephrins are a large family of membrane-bound tyrosine kinase signaling proteins consisting of membrane-bound ligands (ephrins) that interact with complementary receptors (Eph). Ephrin receptors are subdivided into A- and B-class receptors with some interclass non-exclusivity since the EphA4 receptor (expressed on OPCs) can also interact with ephrin B ligands [45,46]. Ephrins ligand–receptor engagement induces bidirectional signaling. Both Eph receptors and ephrin-B ligands become tyrosine phosphorylated through autophosphorylation (receptors) or recruitment of a tyrosine kinase (ligand) [47]. Eph receptors and ephrins are expressed in a variety of CNS diseases and play a role in CNS regeneration in adults by affecting the neural microenvironment [48,49]. Moreover, imbalance of Eph-ephrin function has been implicated in a wide variety of CNS injuries and diseases [50]. Experimental autoimmune encephalitis (EAE) induced in EphA4 receptor knockout mice was shown to inflict a much milder disease and lead to a decreased axonal pathology [51], and ephrin-B1 and B2 knockout were associated with defective Th1 and Th17 differentiation and amelioration of EAE [52]. Several EphA4 receptor inhibitors have been suggested as therapeutic strategies for cancer and several neurological disorders, including MS [53]. The ephrins signaling pathway was shown to have a pivotal role in inhibiting OPCs differentiation [54,55,56,57,58]. Ephrins were found to be expressed on immune cells [52,59,60]. Specifically, ephrins-A1, -A2 and -A3 were shown to be expressed on both CD4^+^ and CD8^+^ developing thymocytes, and it was suggested that this highly compartmentalized expression of ephrin-EphA molecules might affect T cell interactions with stromal cells [59]. Ephrins A1–4 and their receptors Eph A1, A3, A4, A6 and A7, as well as ephrins-B1 and-B2, were identified on immune cells in active MS lesions [52,58,61]. Ephrin-B3 was also identified in MS lesions, and antibody-mediated masking of ephrin-B3 epitopes was shown to promote OPCs differentiation [58]. It was also shown that Eph-ephrin interaction controls the migration of OPCs [62]. Since OPCs differentiation is insufficient in MS lesions, we hypothesized that ephrin expression levels on immune cells of patients with MS may be increased and that this may contribute to the inhibition of OPCs differentiation seen in MS lesions.

Therefore, in this study, we characterized the ephrin expression pattern on immune cell subsets of patients with MS and examined in vitro their signaling effect in a bioassay and on OPCs differentiation.

## 2. Results

### 2.1. The Percentages of Ephrin-A2 and Ephrin-A3-Positive Cells Are Elevated on Immune Cells and Mostly on T Cells of Patients with Relapsing-Remitting Multiple Sclerosis (RR-MS)

We isolated PBMCs from 43 untreated RR-MS patients and 27 age-matched apparently healthy individuals as controls (Table 1).

**Figure 1:**
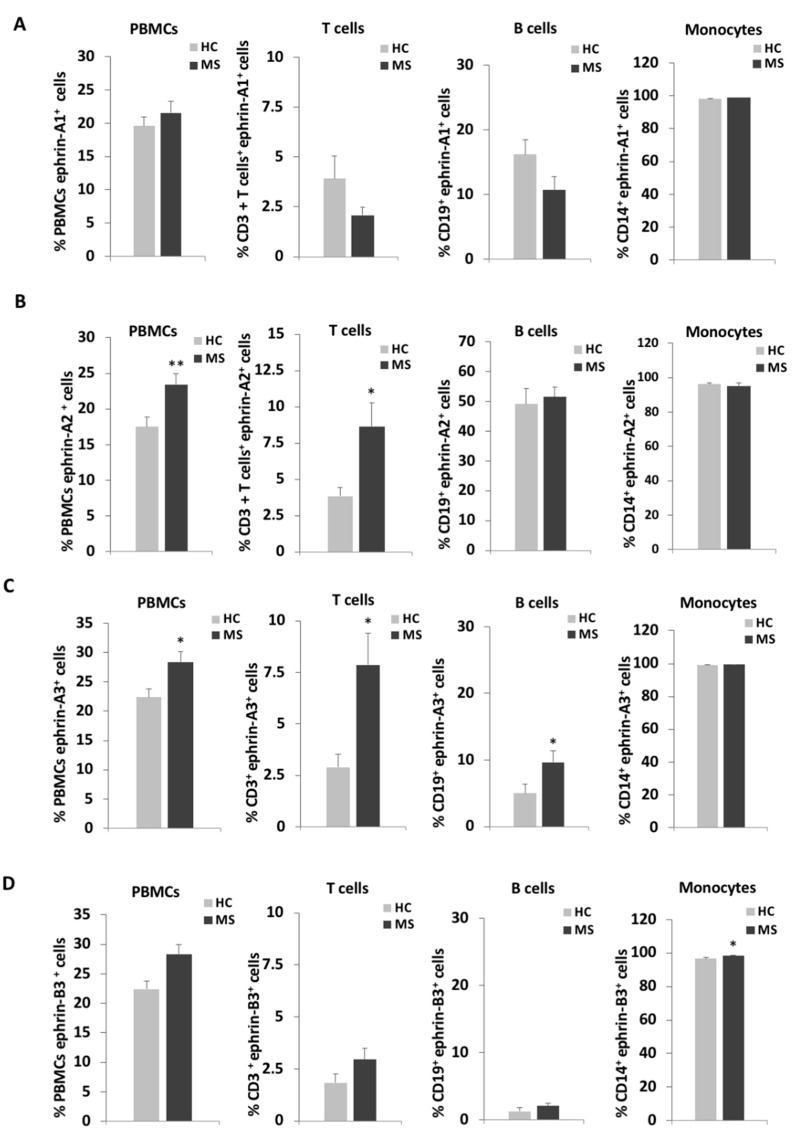
Ephrin expression levels on immune cells of patients with relapsing-remitting multiple sclerosis (RR-MS) and of healthy controls. Immune cells of patients with relapsing-remitting multiple sclerosis (MS) and of healthy controls (HC) were analyzed by flow cytometry for the percentages of expression of ephrin-A1 (**A**), ephrin-A2 (**B**), ephrin-A3 (**C**) and ephrin-B3 (**D**) on total PBMC, CD3^+^ T cells, CD19^+^ B cells and CD14^+^ monocytes. * *p* < 0.05, ** *p* < 0.01.

The PBMCs were incubated with anti-ephrin-A1, -A2, -A3, or -B3 and anti-CD3 (T cells), or anti-CD19 (B cells) or anti-CD14 (monocytes) as well as with fluorochrome-conjugated secondary antibody as described in the Materials and Methods section and in Figure S1A. Cell markers were detected by flow cytometry and analyzed for ephrin expression levels on the immune cells according to the percentage of positive cells for each ephrin (Figure 1; Table S1) and by ephrins’ mean fluorescence intensity (MFI) levels of the immune cells, which indicates the expression density of the tested ephrin on the cells (Table 2). The percentage of ephrin-positive cells and the MFI are complementary measurements for the different aspects of ephrin expression. Overall, ephrin-A1,-A2,-A3, and-B3 were expressed on all of the immunological cells we studied, with almost all of the monocytes expressing these ephrins in healthy individuals and in patients with RR-MS. T cells in general and subpopulations of T cells, as well as B cells, expressed the different ephrins to varying degrees. The percentages of ephrin-A1-positive cells were not significantly different between patients with RR-MS and HC with respect to total PBMCs, T cells, B cells or monocytes (Figure 1A, Table S1). In addition, there was a non-significant trend for the reduction of ephrin-A1 among the T cells of patients with RR-MS. However, its MFI was significantly higher on the T cells of patients with RR-MS than HC (Table 2). The percentages of ephrin-A2-positive cells were significantly higher for PBMCs (23.4 ± 1.5%) and T cells (8.6 ± 1.6%) of patients with RR-MS compared to HC (17.5 ± 1.2%, *p* = 0.006, and 3.8 ± 0.6%, *p* = 0.046, respectively, Figure 1B). The MFI of ephrin-A2 was significantly higher among PBMCs of patients with RR-MS than those of HC (*p* = 0.036, Table 2). The percentages of ephrin-A3-positive cell were significantly higher for total PBMCs (28.4 ± 1.8%), T cells (7.9 ± 1.5%) and B cells (9.6 ± 1.7%) of patients with RR-MS compared to HC (22.4 ± 1.5%, *p* = 0.016; 2.9 ± 0.6%, *p* = 0.025 and 5.0 ± 1.3%, *p* = 0.036, respectively, Figure 1C). The MFI of ephrin-A3 was significantly higher among T cells of patients with RR-MS than those of HC (*p* = 0.010). There was a non-significant trend for increased percentages of ephrin-B3-positive cells among T and B cells of patients with RR-MS (Figure 1D). The MFI of ephrin-B3 in the total number of PBMC cells was significantly higher in patients with RR-MS compared to HC (*p* = 0.022) and B cells (*p* = 0.037).

### 2.2. The Expression of Ephrins-A1, -A2, -A3 and -B3 on T Cell Subpopulations

**Figure 2:**
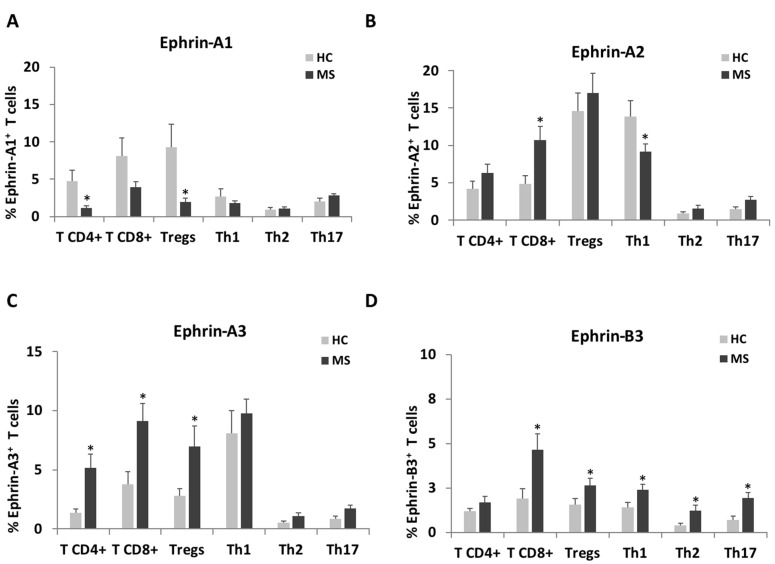
Ephrin expression levels on a subset of T cells of patients with RR-MS patients and of healthy controls. T cells of patients with RR-MS and HC were analyzed by flow cytometry for the percentages of expression of ephrin-A1 (**A**), ephrin-A2 (**B**), ephrin-A3 (**C**) and ephrin-B3 (**D**) on CD4^+^ T cells, CD8^+^ T cells, Tregs, Th1, Th2 and Th17 cells. * *p* < 0.05.

Since the ephrin expression levels were mostly elevated on T cells, we further characterized their expression pattern on specific T cell subtypes (Figure 2, Table 3 and Table S2). CD4^+^ T cells, CD8^+^ T cells, T regulatory (Tregs) cells, T helper (Th)1, Th2 and Th17 cells were detected using flow cytometry extracellular staining as described in Materials and Methods and Figure S1B,C. In accordance with the trend towards a decrease in the percentage of positive ephrine-A1 cells among the total T cells of patients with RR-MS, there were decreased percentages of positive ephrine-A1 cells among CD4^+^ T cells (1.1 ± 0.3%) and Tregs (2.0 ± 0.5%) compared to HC (4.7 ± 1.5%, *p* = 0.028 and 9.3 ± 3.1%, *p* = 0.030, respectively, Figure 2A). However, as with the total T cells, the MFI of ephrin-A1 showed an opposite trend and it was increased in most of the T cell subpopulations and, especially, among Th17 cells (*p* = 0.038, Table 3). As mentioned above, the percentage of ephrin-A2-positive T cells was higher in RR-MS, as manifested by significantly increased percentages of CD8^+^T cells (10.7 ± 1.8%) vs. HC (4.8 ± 1.2%, *p* = 0.008, Figure 2B). There was a trend towards increased percentages of ephrin-A2-positive Tregs (Figure 2B), while the MFI of ephrin-A2 on T-regs was significantly increased among the patients with RR-MS compared to HC (*p* = 0.034). The high percentage of ephrin-A3-positive T cells of patients with RR-MS was mainly due to the significantly high percentage of ephrin-A3-positive CD4^+^ T cells (5.2 ± 1.2%), CD8^+^ T cells (9.1 ± 1.5%) and Tregs (7.0 ± 1.8%) of patients with RR-MS compared to HC (1.4 ± 0.4%, *p* = 0.003; 3.8 ± 1.0%, *p* = 0.005 and 2.8 ± 0.6%, *p* = 0.031, respectively, Figure 2C). Similarly, the MFI of ephrin-A3 was significantly higher on CD4^+^ T cells (*p* < 0.001), CD8^+^ T cells (*p* = 0.042), Tregs (*p* < 0.001) and Th1 cells (*p* = 0.003) of patients with RR-MS compared to HC (Table 3). There was a non-significant trend towards increased ephrin-B3-positive cells among T cells of patients with RR-MS, but the values of CD8^+^ T cells (4.6 ± 0.9%), Tregs (2.6 ± 0.4%), Th1 (2.4 ± 0.3%), Th2 (1.2 ± 0.3%) and Th17 (1.9 ± 0.4%) cells were significantly higher than in HC (1.9 ± 0.6%, *p* = 0.0132; 1.6 ± 0.3%, *p* = 0.050; 1.4 ± 0.3%, *p* = 0.023; 0.4 ± 0.1, *p* = 0.037 and 0.7 ± 0.2, *p* = 0.031, respectively, Figure 2D). In addition, the MFI of ephrin-B3 was significantly higher on Th1 of patients with RR-MS compared to HC (*p* = 0.013).

### 2.3. Ephrins Forward Signaling Is Enhanced When Stimulated with Immune Cells from Patients with RR-MS Compared to Healthy Controls

**Figure 3:**
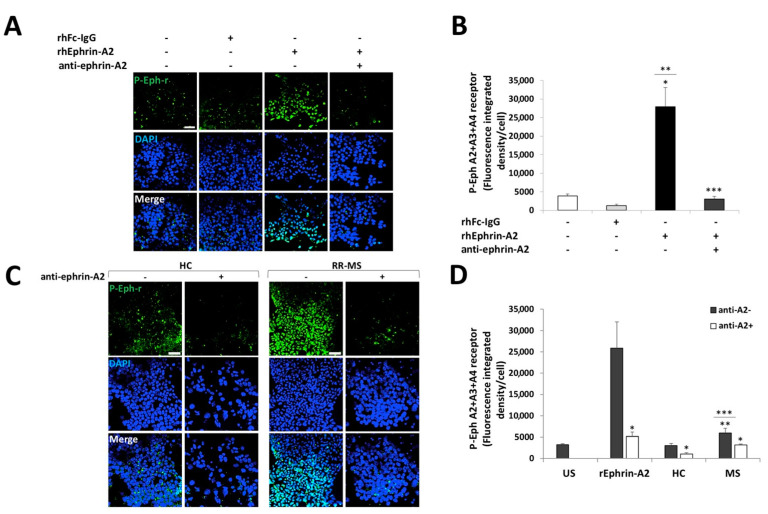
Immune cells of patients with RR-MS increased EphA-receptor phosphorylation in a co-culture bioassay. HEK-293T cells were unstimulated or stimulated with recombinant proteins or co-cultured with immune cells of HC or RR-MS patients and immunostained to phosphorylated Eph A2+A3+A4-receptors (p-Eph-r) and DAPI nuclear staining. (**A**) Representative confocal microscopy images of HEK-293T cells unstimulated or stimulated with 0.5 µg/mL of recombinant human Fc-IgG negative control (hrFc IgG), 0.5 µg/mL human recombinant ephrin-A2 (hrEphrin-A2) protein, and pre-incubated with or without 20 µg/mL anti-ephrin-A2 blocking antibody against ephrin-A2 (anti-ephrin-A2). Magnification ×20; scale = 50 µm. (**B**) Average of the Eph-receptor phosphorylation analysis shown in A. *p* < 0.05, * *p* value of hrEphrin-A2 vs. unstimulated (US), ** *p* value of hrEphrin-A2 vs. hrFc IgG, *** *p* value of hrEphrin-A2 vs. hrEphrin-A2+ anti-ephrin-A2. (**C**) Representative confocal microscope images of immunofluorescence staining for p-Eph-r of 293T-HEK cells co-cultured with immune cells of HC or RR-MS patients −/+ anti-ephrin-A2 antibody. Magnification ×20; scale = 50 µm. (**D**) Average of the Eph-receptor phosphorylation analysis shown in (**C**). * *p* value without vs. with anti-ephrin-A2, ** *p* value of MS vs. US, *** *p* value of MS vs. HC.

In order to examine the biological significance of ephrins overexpression on immune cells of patients with RR-MS, we established a co-culture bioassay for analyzing ephrins forward signaling. 293T-HEK cells, which express Eph-receptors, were starved for 2 h in serum-free medium and then unstimulated or stimulated with recombinant human Fc-IgG negative control fragment (rhFc IgG) or human recombinant Fc-ephrin-A2 (hrEphrin-A2) protein and pre-incubated with or without blocking antibody against ephrin-A2 (anti-ephrin-A2). Following stimulation, the cells were immunostained using a specific antibody to phosphorylated-tyrosine on Eph-A2+A3+A4–receptors (Figure 3A,B). Stimulation with rhEphrin-A2 dramatically increased EphA-receptor phosphorylation, as measured by fluorescence integrated density per cell using ImageJ software (27,953 ± 5116), compared to both unstimulated and rhFc IgG negative control-stimulated cells (3910 ± 565, *p* < 0.001 and 1334 ± 360, *p* = 0.001, respectively, Figure 3B). As expected, pre-incubation of rhEphrin-A2 with the anti-ephrin-A2 blocking antibody almost abolished the phosphorylation enhancement (3023 ± 761, *p* < 0.001, Figure 3B). We also applied this assay for co-culturing 293T-HEK cells with PBMCs from 9 patients with RR-MS or with PBMCs from 10 HC. PBMCs were co-cultured with 293T-HEK cells pre-incubated with or without anti-Ephrin-A2 blocking antibody for stimulation. The PBMCs were then washed out, and 293T-HEK cells were immunostained with the anti-phosphorylated Eph-receptor antibody (Figure 3C,D). A considerable increase in the EphA-receptors phosphorylation was exhibited in the cells that were co-cultured with PBMCs of patients with RR-MS (5932 ± 1141 fluorescence integrated density per cell) compared to HC (3065 ± 479, *p* = 0.041, Figure 3D). Under these conditions, the ephrins levels on the immune cells of patients with RR-MS were sufficient to stimulate Eph-ephrin signaling compared to the culture of 293T-HEK cells alone without PBMCs (3243 ± 244, *p* = 0.048), while ephrin levels on immune cells of HC were not enough to induce EphA-receptors phosphorylation (*p* = 0.746, Figure 3D).

The effect of ephrins-expressing immune cells of patients with RR-MS and HC on forward signaling is demonstrated by the reduction of the extent of the phosphorylation of EphA receptors after adding the anti-ephrin-A2- blocking antibody that was reduced by 47% (3146 ± 254, *p* = 0.014) in patients with RR-MS and by 66% (1045 ± 291, *p* = 0.021) in HC (Figure 3D). These results suggest that ephrin ligands whose expression is increased on immune cells of patients with RR-MS can be directly involved in the enhancement of ephrins forward signaling on cells expressing Eph-receptors, such as OPCs.

### 2.4. Immune Cells of Patients with RR-MS Inhibit the Differentiation of Oligodendrocyte Precursor Cells towards Mature Oligodendrocytes

**Figure 4:**
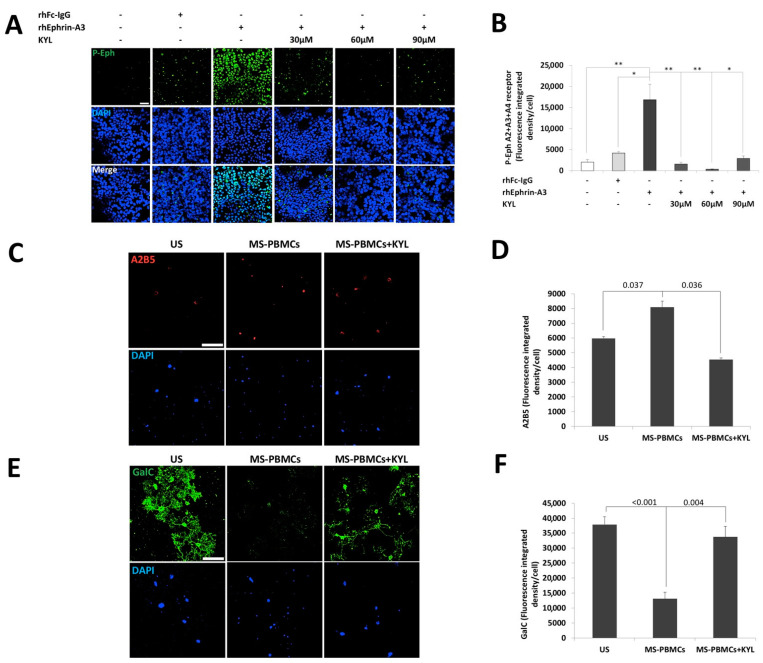
Immune cells of a patient with RR-MS inhibited the differentiation of oligodendrocytes precursor cells to maturing oligodendrocytes. (**A**) Representative confocal microscopy images of HEK-293T cells unstimulated or stimulated with 0.5 µg/mL of recombinant human Fc-IgG negative control (hrFc IgG), or 0.5 µg/mL human recombinant ephrin-A3 (hrEphrin-A3) protein, and pre-incubated with or without 30 µM, 60 µM or 90 µM Eph-receptor inhibitor KYL peptide (KYL) Scheme 20. scale = 50 µm. (**B**) Average of Eph-receptor phosphorylation analysis shown in A. * *p* < 0.05, ** *p* < 0.01. OPCs were unstimulated or co-cultured with PBMCs of patients with RR-MS with or without a pre-incubation with 60 µM inhibitor KYL peptide in oligodendrocyte differentiation medium for 4 days. (**C**) Representative confocal microscopy images of OPCs cells immunostained by anti- A2B5 and DAPI nuclear staining after 4 days of differentiation. Magnification ×25; scale = 50 µm. (**D**) Average of A2B5 expression level analysis. (**E**) Representative confocal microscopy images of OPCs immunostained by GalC and DAPI nuclear staining after 4 days of differentiation. Magnification ×25; scale = 50 µm. (**F**) Average of the GalC expression levels analysis shown in D.

To further investigate the biological effect of ephrins-expressing immune cells of patients with RR-MS, we studied their impact on oligodendrocytes precursor cells (OPCs) differentiation (Figure 4). Given that several ephrins were found to be overexpressed on immune cells of patients with RR-MS, we used a synthetic peptide (KYL) that blocks all the ephrin ligands interactions with EphA4 receptors [63]. We first studied the effect of KYL on the forward signaling in 293T-HEK cells (Figure 4A,B). As expected, the KYL peptide significantly reduced the Eph-receptor phosphorylation, with the optimal concentration of 60 µM KYL (fluorescence-integrated density/cell 320 ± 111) compared to recombinant human ephrin-A3 protein (rhEphrin-A3) alone (16,836 ± 3738, *p* = 0.010, Figure 4B). We then co-cultured primary OPCs with PBMCs of patients with RR-MS, with or without pre-incubation, with the Eph-receptor inhibitor KYL peptide. Following four days of differentiation, the PBMCs were washed out, and the OPC-derived cells were immunostained for A2B5, an undifferentiated OPCs marker (Figure 4C,D), and for GalC, a mature myelinating oligodendrocytes marker (Figure 4E,F). Interestingly, A2B5 expression levels were significantly higher in the cells that were co-cultured with the PBMCs of the patient with RR-MS (2647 ± 395 fluorescence integrated density/cell) compared to the cells that were cultured with the differentiation condition alone (810 ± 111, *p* = 0.003), and to the cells that were co-cultured with PBMCs and the KYL inhibitor (1078 ± 108, *p* = 0.007, Figure 4D). GalC was highly expressed in the cells that were cultured with the differentiation condition alone (37,902 ± 2521 fluorescence-integrated density/cell) that also showed an increased branching morphology of the differentiating cells (Figure 4E). GalC expression was significantly reduced in cells that were co-cultured with the PBMCs of the patient with RR-MS (13,069 ± 2225, *p* < 0.001), and it also showed a much lower branched morphology that reflected less differentiation of the OPCs. Moreover, this inhibition of OPCs differentiation by PBMCs from the same patient was nearly eliminated when the cells were pre-incubated with the KYL inhibitor (33,744 ± 3574, *p* = 0.004, Figure 4F). In conclusion, it would appear that ephrin expression on the immune cells of RR-MS patients may contribute to inhibition of OPCs differentiation.

## 3. Discussion

Spontaneous remyelination of MS lesions is insufficient when there is a failure of adult OPCs to differentiate into mature myelinating oligodendrocytes [64]. MS is an immune-mediated disease that causes demyelination as the main pathological feature of tissue damage. It is, therefore, important to understand the effect of inflammatory activity on the regenerative capacity of oligodendrocytes in MS. The innate and adaptive immune responses were found to have different trends in their effect on the regeneration of oligodendrocytes [26,27,28,65,66]. In addition, the effects of T cell subgroups and of secreted immunological factors on oligodendrocyte differentiation have been investigated [29,30,31,32,33,34,38,67]. We report here about ephrins, which are a membrane-bound family of proteins that act via cell contact interaction, inhibit OPCs differentiation and have been identified in MS lesions. Ephrin-A1-4, Eph-A1, -A3, -A4, -A6 and -A7 receptors are expressed on perivascular mononuclear inflammatory cells, reactive astrocytes and macrophages in active MS lesions [61]. Ephrin-B3 expression was also demonstrated in extracts from MS lesions [58], and foamy macrophages within active MS lesions have shown broad ephrin/Eph expression, suggesting their involvement in the pathology of the disease [61]. We compared the ephrin expression on immune cells, their signaling effect between patients with RR-MS and HC, as well as their effect on the differentiation of OPCs. As far as we know, this is the first description of such an extensive characterization of ephrin expression patterns on immune cells of patients with RR-MS.

Our findings revealed that ephrins-A1, -A2, -A3 and -B3 are expressed on peripheral blood immune cells of healthy individuals as well as on those of patients with RR-MS. Specifically, we found an increased expression of ephrins-A2, -A3 and -B3 mainly on the T cells of patients with RR-MS, suggesting an activity that inhibits oligodendrocyte differentiation. Indeed, T cells have been reported to inhibit remyelination by targeting OPCs [68]. Our major findings were that the percentages of ephrins-A2, -A3 and -B3 positive cells were significantly higher on the CD8^+^ T cells of patients with RR-MS and that there was also an increase in the MFI of ephrin-A3 on CD8^+^ T cells. Of note, cytotoxic CD8^+^ T cells are often found in close proximity to oligodendrocytes and demyelinated axons in MS [69]. Additionally, oligodendrocytes presented antigen and activate CD8^+^ T cells in an EAE model, and adoptive transfer of myelin-reactive T cells resulted in reduced numbers of oligodendrocytes and reduced remyelination [70]. OPCs that were exposed in vitro to IFNγ, cross-presented antigens to cytotoxic CD8^+^ T cells that led to OPCs death [68]. CD8^+^ T cells were originally considered to exert a suppressive role in demyelinating disease. However, there is growing evidence that supports a pathogenic role of CD8^+^ T cells in MS [37,71,72,73,74]. The increased ephrin-A2, -A3 and -B3 expressions on CD8^+^ T cells suggest that infiltrating CD8+ T cells in the MS lesion and the meninges may contribute to the inhibition of OPCs differentiation into myelinating oligodendrocytes. Higher percentages of ephrin-A3 and -B3 positive cells were found on Tregs of patients with RR-MS, while high MFIs of ephrin-A3 and -B3 were found on Th1 cells. Of the ephrins tested in the current investigation, ephrin-A3 and, to a lesser extent, ephrin-B3 was the most highly expressed on the various immune cells, especially the T cells subtypes, of patients with RR-MS.

Both ephrin-A3 and -B3 were substantially elevated on Tregs cells. Several recent studies have suggested the importance of Tregs in promoting remyelination [34,75]. Tregs may exert an oligodendroglia regenerative effect via CCN3 that promotes oligodendrocyte differentiation and myelination [33]. However, the overexpression of ephrins on the Tregs of patients with MS may impair their positive effect on oligodendrocyte differentiation and myelination. In this context, it is worth noting that Tregs that were isolated from MS patients were found to have a defective regulatory function on T cell activity [75].

The highly expressed ephrin-A3 on immune cells of patients with RR-MSwas found to be a putative target of miR-210 for downregulation [76,77]. Interestingly, remyelination was much more extensive in tissues caudal to injured spinal cord sites of mice injected with miR-210 [76]. MiR-210 was also shown to affect the myelin in the peripheral nerves by increasing both the proliferation and migration of Schwann cells to the injury site and the expression of myelin basic protein [78].

Ephrin-B3, which was also elevated on CD8^+^ T cells, Tregs and Th1 cells of patients with RR-MS, is considered a physiologically important myelin-associated inhibitor of axonal growth in the adult central nervous system [79]. Ephrin-B3-EphB3 interactions were shown to function as mediators of oligodendrocyte cell death following contusive spinal cord injury [80]. Furthermore, OPCs failed to differentiate in vitro in the presence of ephrin-B3, and infusion of ephrin-B3 inhibited remyelination in a rat model while masking ephrin-B3 epitope-promoted remyelination [58].

Collectively, our results demonstrated that ephrins were overexpressed on the immune cells of patients with RR-MS, that they increased the EphA-receptor phosphorylation for enhanced forward signaling of ephrins, and that they inhibited OPCs differentiation into mature oligodendrocytes. They also suggested that the increased expressions of ephrins, especially of ephrin-A3, on CD8^+^ T and Treg cells contribute to the inhibition of OPCs differentiation present in MS lesions and to the inadequate repair of the demyelinating damage of the MS disease process.

## 4. Materials and Methods

### 4.1. Study Population

Patients with relapsing-remitting multiple sclerosis (RR-MS) attending the Neuroimmunology Clinic at the Tel Aviv Sourasky Medical Center were included in the study, and age- and sex-matched apparently healthy individuals comprised the control group. All of the patients were untreated for a minimum period of 3 months during clinical remission. Blood samples were drawn from 43 untreated patients with RR-MS and 27 healthy controls (Table 1). All experiments were approved by the institutional ethics committee, and informed consent was obtained from all participants.

### 4.2. Cell Collection and Culture

Peripheral blood mononuclear cells (PBMCs) were isolated from heparinized venous blood by centrifugation over Ficoll-Paque (Lymphoprep; Alere Technologies AS, Oslo, Norway). The cells were resuspended in a freezing solution containing 10% dimethyl sulfoxide (DMSO) (Sigma, St. Louis, MO, USA) and 90% fetal bovine serum (FBS) (Biological Industries, Kibbutz Beit Hemek, Israel) and were frozen at −80 °C in an iso-propanol-jacketed closed container overnight. The cells were then stored in liquid nitrogen until use. A human embryonic kidney (HEK-293T) cell line (ATCC, Manassas, VA, USA) was maintained in DMEM (low glucose) medium supplemented with 10% FBS, 4 mM L-glutamine, 50 units/mL penicillin, and 50 µg/mL streptomycin (Biological Industries, Kibbutz Beit Hemek, Israel). The cells were cultured at 37 °C in a humidified atmosphere and 5% CO_2_ in the air.

### 4.3. Flow Cytometry

PBMCs were thawed and resuspended (1 × 10^6^ cells/mL) in phosphate buffer saline (PBS). Dead cells were stained with ViviD (fixable violet; Invitrogen, Eugene, OR, USA) according to the manufacturer’s protocol. Following washing, the cells were incubated with rabbit anti-human ephrin-A1 (Thermo Fisher Scientific, Rockford, IL, USA), mouse anti-human ephrin-A2 (Novus Bioscience, Centinal, CO, USA), rabbit human ephrin-A3 (LSBio, Seattle, WA, USA) or rabbit anti-human ephrin-B3 (Novus Bioscience) in blocking buffer containing 3% bovine serum albumin (BSA) in PBS for 30 min at 4 °C, washed and stained indirectly with fluorochrome-conjugated PE-anti-mouse/PE-anti-rabbit IgG (ab’)_2_ fragments (Jackson ImmunoResearch, Avondale, PA, USA) in blocking buffer for 30 min at 4 °C. The cells were subsequently co-stained with fluorochrome-conjugated mouse monoclonal antibodies against human CD3 (AF780; eBioscience, San Diego, CA, USA), CD4 (PE-Cy5.5; eBioscience), CD8 (BV650; BD Biosciences, San Jose, CA, USA), CD19 (APC; BD Biosciences) and CD14 (FITC; Milteny Biotec, Bergisch Gladbach, Germany) among the total PBMCs; CD3, CD4,CD25 (FITC; eBioscience) and CD127 (APC; eBioscience) for T-regulatory (Treg) cells; and CD3, CD4, CXCR3 (Alexa Flour488, BioLegend, San Diego, CA, USA), CCR4 (PE/Cy7; BioLegend), CCR6 (BV650; BD Bioscience) and CCR10 (APC; BioLegend) for T-helper (Th) cell subsets of Th1, Th2 and Th17. Flow cytometry was performed on a BD CantoII flow-cytometer (BD Biosciences), and samples were analyzed with FlowJo software (FlowJo LLC; Becton Dickinson, Ashland, Oregon). The cells were gated as follows: human PBMCs (T cells CD3^+^, CD4^+^or CD8^+^, B cells CD19^+^ and monocytes CD14^+^) (Figure S1A), Treg cells (CD4^+^ CD127^−^CD25^+high^) according to OMIP-15 [81] (Figure S1B) and Th cells (CD4^+^CCR4^−^CXCR3^+^ -Th1; CD4^+^CCR6^−^CCR4^+^CXCR3^−^ CCR10^-^ -Th2; CD4^+^CCR6^+^CCR4^+^CXCR3^−^ CCR10^-^ -Th17) according to OMIP-17 [82] (Figure S1C). The detected parameters were the percentage of ephrin positive cells for each of the different cell types and the ephrins mean fluorescence intensity (MFI) on these cells.

### 4.4. Ephrin Phosphorylation In-Vitro Assay

HEK-293T (HEK) cells were seeded on 0.01% poly-l-lysine (PLL)-coated (Sigma, St. Louis, MO, USA) glass coverslips (0.33 cm^2^) in a 24-well culture plate 2 days before stimulation. The cells were harvested in a DMEM serum-free (DMEM-SF) medium supplemented with glutamine and antibiotics, as mentioned above. Recombinant human Fc-IgG fragments or ephrinA2-Fc fragments (R&D, Minneapolis, MN, USA) were mixed with or without anti-ephrin-A2 blocking antibody (AF607; R&D) and incubated at 37 °C for 1 h prior to stimulation. The competitive ephrin inhibitor peptide KYLPYWPVLSSL (KYL) (Tocris Bioscience, Abingdon, UK) was added to the cells in DMEM-SF medium 30 min before stimulation.

For co-culture, the PBMCs were thawed and recovered by resuspension in a complete culture medium comprised of RPMI-1640 medium supplemented with 10% FBS, 4 mM l-glutamine, 50 units/mL penicillin, and 50 µg/mL streptomycin (Biological Industries) and incubated at 37 °C humidified atmosphere and 5% CO_2_ in the air for at least 2 h. Ephrin stimulation was carried by incubating the HEK cells with the recombinant protein with or without a blocking antibody or in co-culture with 10^6^ recovered PBMCs in DMEM-SF medium for 30 min at 37 °C. Following stimulation, the cells were washed with cold PBS and fixed with 4% paraformaldehyde solution (PFA) for 15 min at room temperature and subjected to immunofluorescence staining.

### 4.5. Oligodendrocyte Precursor Cells Differentiation

Rat glial precursor cells (RPCs/OPCs) were purchased from Invitrogen (Eugene, OR, USA) and handled according to the manufacture’s protocol. Briefly, the cells were thawed and cultured on flasks coated with 10 µg/mL poly-l-ornithine (Sigma) at a seeding density of 3 × 10^4^ cells per cm^2^. Oligodendrocyte precursor cells (OPCs) were expanded for about 2 weeks on KnockOut^tm^ Dulbecco’s Modified Eagle’s medium/F12 (KO-DMEM/F12) medium containing: 2 mM GlutaMAX^TM^ -I supplement, 1 × N-2 supplement, 1 × B-27 (Gibco, Grand Island, NY, USA), 20 ng/mL bFGF, 20 ng/mL EGF, 10 ng/mL PDGF-AA (Peprotech, Rocky Hill, NJ, USA) and 10 ng/mL penicillin/streptomycin antibiotics (Biological Industries). The medium was replaced every other day. For oligodendrocyte differentiation, the cells were transferred to 10 µg/mL poly-l-ornithine (Sigma) and laminin-coated (Invitrogen) glass coverslips (0.33 cm^2^) in a 24-well culture plate. The cells were expanded for two days with the same medium and then switched to differentiating medium without serum or in co-culture with 1 × 10^6^ PBMCs. PBMCs were pre-recovered with a complete RPMI medium in a 37 °C humidified incubator for 2 h. KYL Inhibitor peptide (Tocris Bioscience) was added to the cells 30 min before co-culturing with PBMCs in a differentiation medium. The differentiation medium contained KO-DMEM/F12 with 2 mM GlutaMAX^TM^ -I supplement, 1 × N-2 supplement, 1 × B-2 supplement, 5 µg/mL insulin, 5 µg/mL *N*-acetyl-l-cysteine (Sigma), 0.1% BSA (Millipore, Kankakee, IL, USA), 2 ng/mL BDNF, 2 ng/mL CNTF (Peprotech) and 10 ng/mL antibiotics. The cells were cultured in differentiating medium for 4 days, and three-quarters of the medium was replaced every other day. After differentiation, the cells were fixed and subjected to immunofluorescence staining.

### 4.6. Immunofluorescence Staining and Confocal Analysis

The cells were washed with cold PBS and fixed for 15 min at room temperature in 4% paraformaldehyde (PFA)/PBS, washed three times with PBS, and permeabilized for 3 min in 0.2% Triton X-100/PBS (for intracellular markers only). Blocking was done in 1% BSA/10% normal donkey serum/PBS for 30 min at room temperature. The cells were subsequently incubated with the primary antibodies against EphA2 + A3 + A4 receptor phospho Y588 + Y596 (1:50; Abcam, Cambridge, MA, USA), A2B5 (1:100; Invitrogen) or GalC (1:100; EMD Millipore) diluted in primary antibody dilution buffer (Biomeda Corporation, Foster City, CA, USA) and incubated for 2 h at room temperature or overnight at 4 °C. The cells were washed three times with PBS and then incubated with secondary antibodies (Alexa Flour-488 donkey-anti-rabbit, Alexa-549- donkey anti-mouse IgM or Alexa Flour-488 donkey-anti-mouse IgG; Invitrogen) diluted 1:200 in 5% NDS/PBS for 1 h in the dark. They were then stained with 1 µg/mL DAPI (Sigma) in PBS for 5 min. The cells were washed in PBS, and the cover glasses were mounted onto Histobond slides using Immuno-Mount (Thermo Scientific, Loughborough, UK) and imaged by a Zeiss LSM 710 confocal microscope. Identical parameters (e.g., scanning line, laser light, contrast, and brightness) were used for comparing fluorescence intensities of the different conditions. Between 5–8 microscopic fields were taken from each sample, and a representative field is shown in the figures. Image analysis was performed with ImageJ software (NIH, Bethesda, MD, USA)). DAPI staining was used to define the nuclear region and the number of cells per field. Quantitative fluorescence data were exported from ImageJ-generated histograms into Microsoft Excel software for further analysis and presentation. The fluorescence integrated density of each field was divided into cell numbers of the same field. The mean fluorescence integrated density per cell was quantified from 5–8 different fields and under the different study groups, after which it was calculated and compared.

### 4.7. Statistical Analysis

The data are expressed as an average of the means ± SEM. Student’s *t*-test was used to compare differences between the study groups. Statistical significance was set at *p* < 0.05.

## Figures and Tables

**Table 1:** Clinical data summary.

	Untreated RR-MS Patients	Healthy Controls
Number	43	27
Males	18	8
Females	25	19
Age (mean ± SD), years	37.7 ± 11.86	38.9 ± 9.52
Range, years	(19–69)	(19–60)
EDSS (mean ± SD)	2.15 ± 2.16	
Range	(0–7)	
Disease duration, years (mean ± SD)	6.79 ± 7.61	
Range, years	(0–28)	

**Table 2:** Ephrin means fluorescence intensity on different immune cells.

	Ephrin-A1			Ephrin-A2			Ephrin-A3			Ephrin-B3		
	HC	MS	*p*	HC	MS	*p*	HC	MS	*p*	HC	MS	*p*
**PBMCs**	7305 ± 563	8630 ± 970	0.133	6968 ± 599	8873 ± 958	**0.036**	10,713 ± 1199	13,585 ± 1227	0.053	5974 ± 573	8035 ± 972	**0.022**
**T cells**	8395 ± 1430	15,252 ± 1905	**0.005**	6649 ± 1016	6517 ± 406	0.905	6323 ± 858	9340 ± 711	**0.010**	3540 ± 478	3950 ± 267	0.458
**B cells**	5434 ± 832	5670 ± 771	0.836	8314 ± 1002	7403 ± 521	0.440	5792 ± 781	6226 ± 890	0.715	3610 ± 514	6051 ± 1037	**0.037**
**Mo**	7852 ± 779	8926 ± 642	0.272	8088 ± 917	9855 ± 821	0.132	11,121 ± 1437	13,899 ± 1082	0.106	6367 ± 794	8614 ± 740	**0.033**

PBMCs—peripheral mononuclear cells; Mo—monocytes.

**Table 3:** Ephrin mean fluorescence intensity on different T cell subtypes.

	Ephrin-A1			Ephrin-A2			Ephrin-A3			Ephrin-B3		
	HC	MS	*p*	HC	MS	*p*	HC	MS	*p*	HC	MS	*p*
**CD4+**	5623 ± 1538	8800 ± 1498	0.144	5699 ± 733	5804 ± 350	0.898	5669 ± 739	9422 ± 766	**<0.001**	2608 ± 326	3118 ± 225	0.196
**CD8+**	1138 ± 80	1211 ± 80	0.518	2476 ± 253	2577 ± 162	0.739	3237 ± 547	4912 ± 596	**0.042**	2020 ± 216	1709 ± 118	0.213
**T-regs**	1200 ± 155	1793 ± 261	0.055	2973 ± 231	4186 ± 510	**0.034**	2414 ± 377	4537 ± 465	**<0.001**	1201 ± 147	1719 ± 243	0.076
**Th1**	582 ± 87	731 ± 134	0.357	710 ± 48	826 ± 88	0.254	661 ± 71	1004 ± 85	**0.003**	401 ± 21	514 ± 39	**0.013**
**Th2**	676 ± 75	3256 ± 1353	0.065	660 ± 96	1070 ± 414	0.340	846 ± 87	1124 ± 133	0.078	540 ± 72	685 ± 60	0.117
**Th17**	4268 ± 1870	22,841 ± 8471	**0.038**	882 ± 149	824 ± 149	0.785	971 ± 166	838 ± 118	0.518	503 ± 74	728 ± 159	0.207

Treg—T regulatory cells. Th—T helper cells. Bold values indicate significant differences.

## Data Availability

The data presented in this study are available on request from the corresponding author. The data are not publicly available due to ethical restrictions.

## Supplementary Materials

The following are available online at https://www.mdpi.com/1422-0067/22/4/2182/s1, Figure S1: FACS gating and analysis of ephrins on immune cells., Table S1: % Ephrins expression different Immune cells. Table S2: % Ephrins expression on different T cells.
