# Supplementary material for: Increased Expression of Ephrins on Immune Cells of Patients with Relapsing Remitting Multiple Sclerosis Affects Oligodendrocyte Differentiation
Source: Int J Mol Sci. 2021 Feb 22;22(4):2182. doi: 10.3390/ijms22042182 (PMC7927032; doi:10.3390/ijms22042182)
Supplement: Supplementary file 1 [file ijms-22-02182-s001.pdf]

**A**

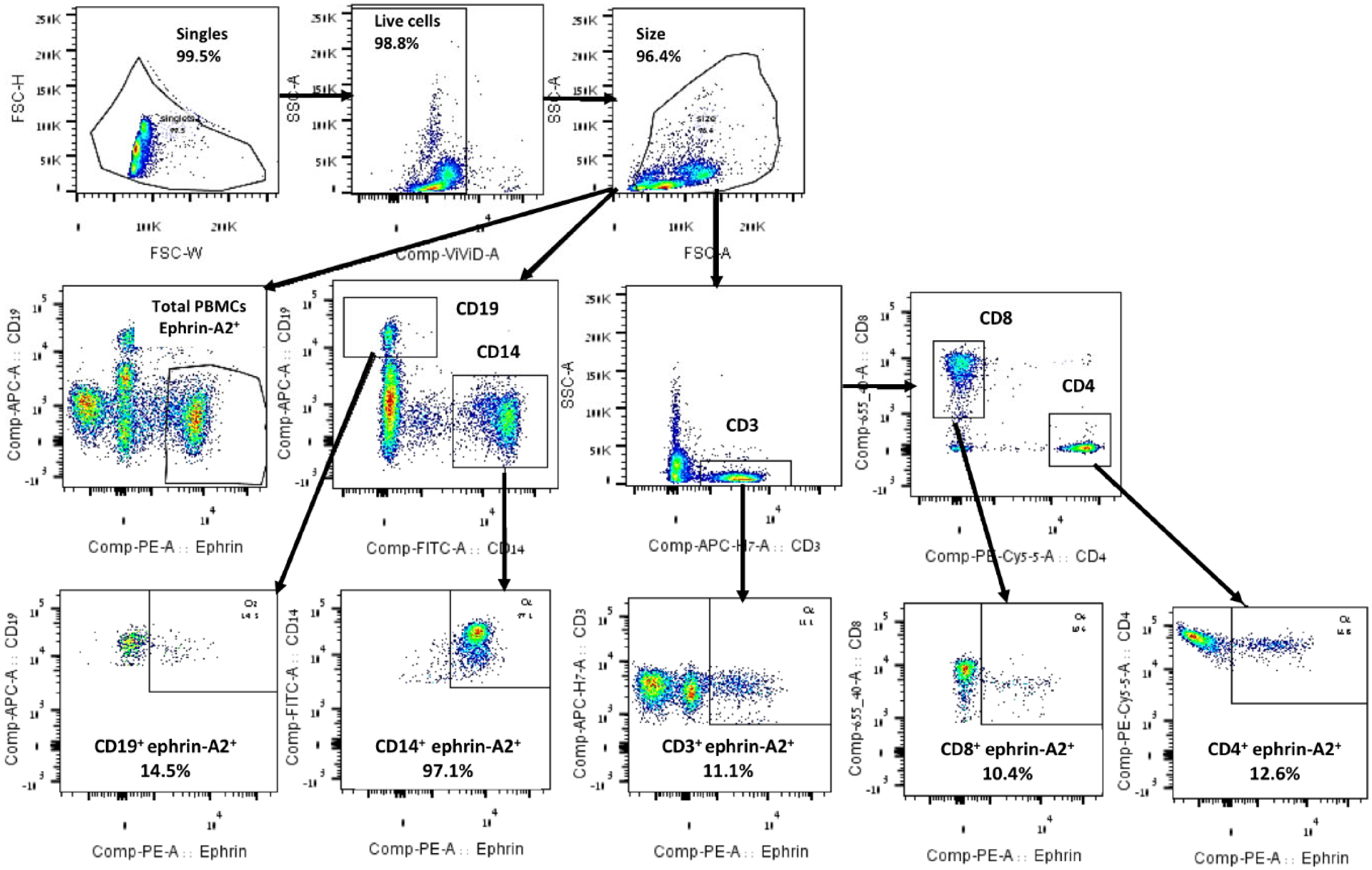

Singles
99.5%
FSC-H
FSC-W
Live cells
98.8%
SSC-A
Comp-ViViD-A
Size
96.4%
SSC-A
FSC-A
Total PBMCs
Ephrin-A2+
Comp-APC-A :: CD19
Comp-PE-A :: Ephrin
CD19
CD14
Comp-APC-A :: CD19
Comp-FITC-A :: CD14
CD3
SSC-A
Comp-APC-H7-A :: CD3
CD8
CD4
Comp-PE-Cy5-5-A :: CD4
CD19+ ephrin-A2+
14.5%
Comp-APC-A :: CD19
Comp-PE-A :: Ephrin
CD14+ ephrin-A2+
97.1%
Comp-FITC-A :: CD14
Comp-PE-A :: Ephrin
CD3+ ephrin-A2+
11.1%
Comp-APC-H7-A :: CD3
Comp-PE-A :: Ephrin
CD8+ ephrin-A2+
10.4%
Comp-655_40-A :: CD8
Comp-PE-A :: Ephrin
CD4+ ephrin-A2+
12.6%
Comp-PE-Cy5-5-A :: CD4
Comp-PE-A :: Ephrin

**B**

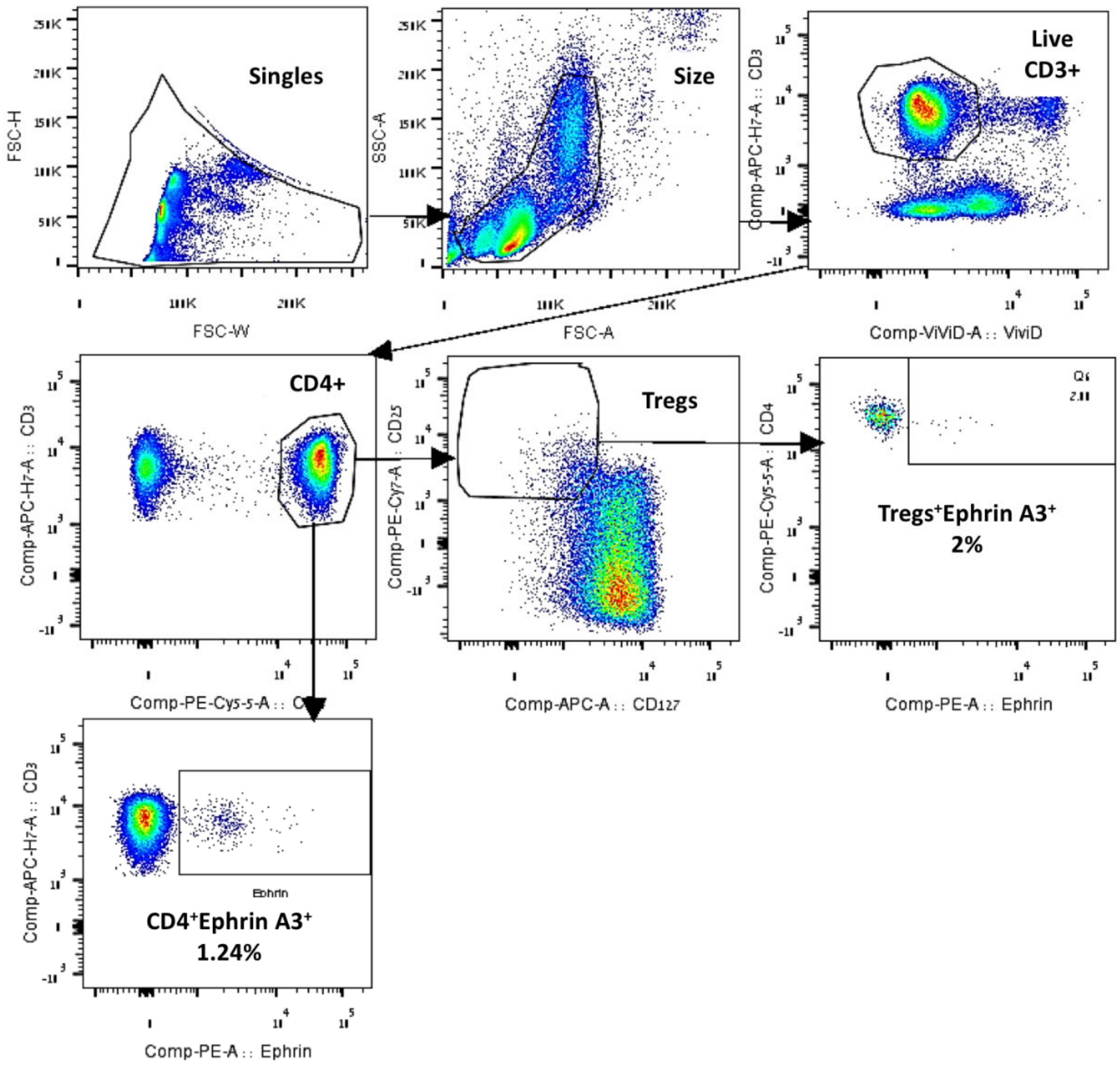
Singles
FSC-H
FSC-W
Size
SSC-A
FSC-A
Live
CD3+
Comp-APC-H7-A :: CD3
Comp-ViViD-A :: ViviD
CD4+
Comp-APC-H7-A :: CD3
Comp-PE-Cy5-5-A :: C
Tregs
Comp-PE-Cy7-A :: CD25
Comp-APC-A :: CD127
Tregs+Ephrin A3+
2%
Comp-PE-Cy5-5-A :: CD4
Comp-PE-A :: Ephrin
Ephrin
CD4+Ephrin A3+
1.24%
Comp-APC-H7-A :: CD3
Comp-PE-A :: Ephrin

C

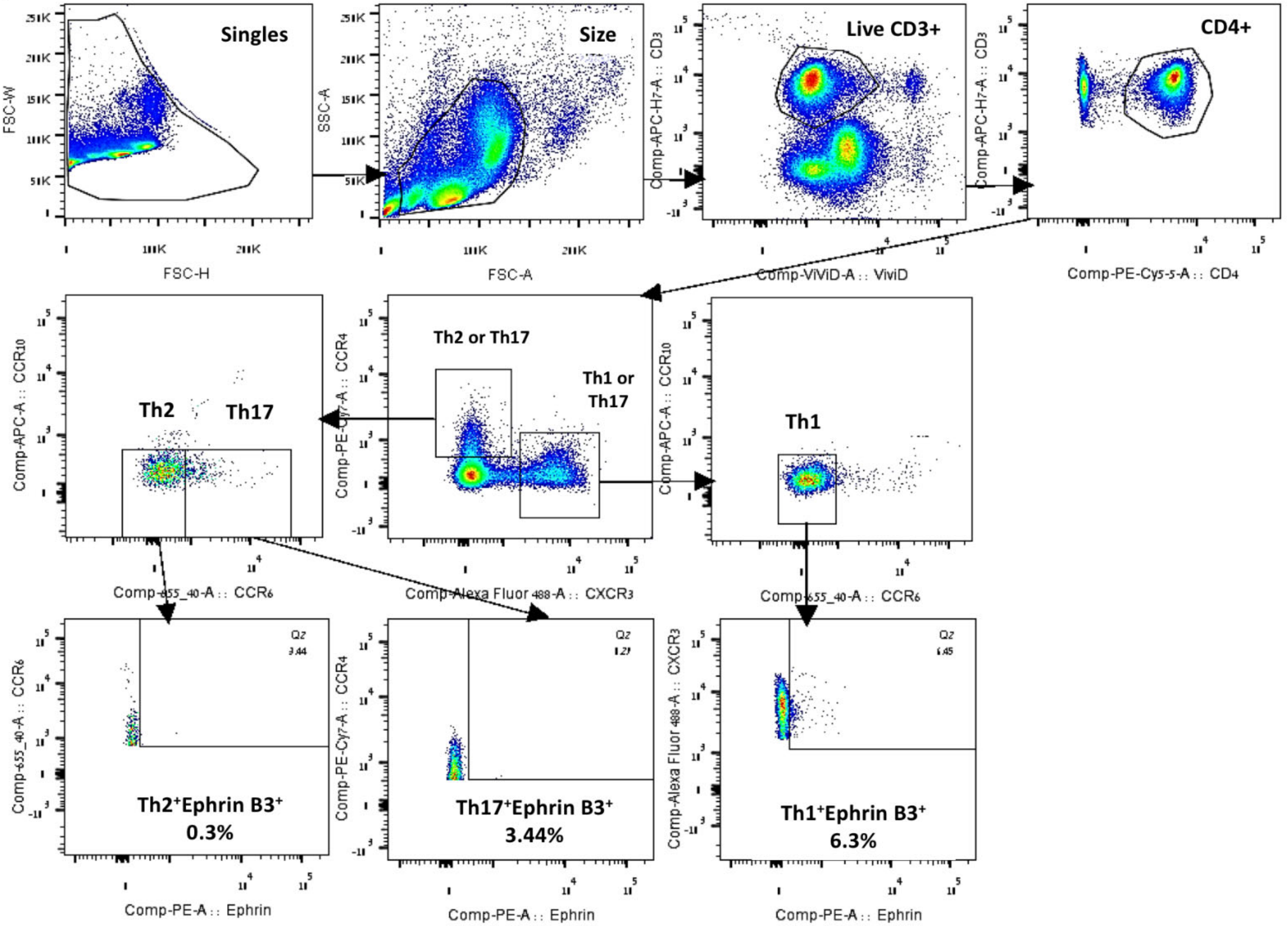
Singles
FSC-W
FSC-H
Size
SSC-A
FSC-A
Live CD3+
Comp-APC-H7-A :: CD3
Comp-ViViD-A :: ViviD
CD4+
Comp-APC-H7-A :: CD3
Comp-PE-Cy5-5-A :: CD4
Th2
Th17
Comp-APC-A :: CCR10
Comp-655_40-A :: CCR6
Th2 or Th17
Th1 or Th17
Comp-PE-Cy7-A :: CCR4
Comp-Alexa Fluor 488-A :: CXCR3
Th1
Comp-APC-A :: CCR10
Comp-655_40-A :: CCR6
Th2+Ephrin B3+
0.3%
Comp-655_40-A :: CCR6
Comp-PE-A :: Ephrin
Th17+Ephrin B3+
3.44%
Comp-PE-Cy7-A :: CCR4
Comp-PE-A :: Ephrin
Th1+Ephrin B3+
6.3%
Comp-Alexa Fluor 488-A :: CXCR3
Comp-PE-A :: Ephrin

**Figure S1:** FACS gating and analysis of ephrins on immune cells. (**A**) A representative FACS gating and analysis for measuring the %ephrin-A2$^{+}$ PBMCs and the among different immune cells: monocytes (CD14$^{+}$), B cells (CD19$^{+}$), T cells (CD3$^{+}$), CD4$^{+}$ T cells and CD8$^{+}$ T cells. (**B**) A representative FACS gating and analysis for %ephrin-A3$^{+}$ T regulatory (Tregs) cells (CD4$^{+}$ CD127$^{-}$CD25$^{high}$). (**C**) A representative FACS gating and analysis of %ephrin-B3$^{+}$ T helper (Th) cells: Th1 (CD4$^{+}$CXCR3$^{+}$), Th2 (CD4$^{+}$CCR4$^{+}$CCR6$^{-}$) and Th17 (CD4$^{+}$CCR4$^{+}$CCR6$^{+}$). MFI was calculated by ImageJ for the ephrin positive cells.

**Table S1:** % Ephrins expression on different Immune cells.

| | **Ephrin-A1** | | | **Ephrin-A2** | | | **Ephrin-A3** | | | **Ephrin-B3** | | |
|---|---|---|---|---|---|---|---|---|---|---|---|---|
| | HC | MS | *p* | HC | MS | *p* | HC | MS | *p* | HC | MS | *p* |
| **PBMCs** | 19.6±1.4 | 21.6±1.8 | 0.406 | 17.5±1.2 | 23.4±1.5 | **0.006** | 22.4±1.5 | 28.4±1.8 | **0.016** | 19.3±1.3 | 22.5±1.6 | 0.155 |
| **T cells** | 3.9±1.1 | 2.1±0.4 | 0.080 | 3.8±0.6 | 8.6±1.6 | **0.046** | 2.9±0.6 | 7.9±1.5 | **0.025** | 1.8±0.4 | 3±0.5 | 0.172 |
| **B Cells** | 16.2±2.3 | 10.7±2.1 | 0.078 | 49.1±5.3 | 51.5±3.4 | 0.709 | 5±1.3 | 9.6±1.7 | **0.036** | 1.3±0.5 | 2.1±0.4 | 0.232 |
| **Mo** | 98±0.5 | 98.7±0.2 | 0.177 | 96.2±0.8 | 95.2±1.9 | 0.637 | 99.1±0.2 | 99.4±0.1 | 0.175 | 96.6±0.7 | 98.4±0.3 | **0.032** |

PBMCs, Total peripheral blood mononuclear cells; Mo, Monocytes; Average ±S.E.M.

**Table S1:** % Ephrins expression on different T cells.

| | **Ephrin-A1** | | | **Ephrin-A2** | | | **Ephrin-A3** | | | **Ephrin-B3** | | |
|---|---|---|---|---|---|---|---|---|---|---|---|---|
| | HC | MS | *p* | HC | MS | *p* | HC | MS | *p* | HC | MS | *p* |
| **CD4+** | 4.7±1.5 | 1.1±0.3 | **0.028** | 4.2±1 | 6.3±1.1 | 0.162 | 1.4±0.4 | 5.2±1.2 | **0.003** | 1.2±0.2 | 1.7±0.3 | 0.166 |
| **CD8+** | 8.1±2.4 | 4±0.7 | 0.109 | 4.8±1.2 | 10.7±1.8 | **0.008** | 3.8±1 | 9.1±1.5 | **0.005** | 1.9±0.6 | 4.6±0.9 | **0.013** |
| **Tregs** | 9.3±3.1 | 2±0.5 | **0.030** | 14.6±2.4 | 17±2.6 | 0.493 | 2.8±0.6 | 7±1.8 | **0.031** | 1.6±0.3 | 2.6±0.4 | 0.050 |
| **Th1** | 2.7±1.1 | 1.8±0.3 | 0.374 | 13.8±2.1 | 9.2±1.1 | **0.035** | 8.1±1.9 | 9.8±1.2 | 0.446 | 1.4±0.3 | 2.4±0.3 | **0.023** |
| **Th2** | 1±0.2 | 1.1±0.3 | 0.823 | 0.9±0.2 | 1.6±0.4 | 0.209 | 0.5±0.1 | 1.1±0.3 | 0.123 | 0.4±0.1 | 1.2±0.3 | **0.037** |
| **Th17** | 2±0.5 | 2.8±0.7 | 0.408 | 1.5±0.3 | 2.7±0.6 | 0.136 | 0.9±0.2 | 1.7±0.4 | 0.106 | 0.7±0.2 | 1.9±0.4 | **0.031** |

T-regs, T regulatory cells; Th , T helper cells; Average ±S.E.M.
